# Sanguinarine Exhibits Antiviral Activity against Porcine Reproductive and Respiratory Syndrome Virus via Multisite Inhibition Mechanisms

**DOI:** 10.3390/v15030688

**Published:** 2023-03-06

**Authors:** Qiyun Ke, Kaiqi Duan, Yan Cheng, Si Xu, Shaobo Xiao, Liurong Fang

**Affiliations:** 1 State Key Laboratory of Agricultural Microbiology, College of Veterinary Medicine, Huazhong Agricultural University, Wuhan 430070, China; 2 The Key Laboratory of Preventive Veterinary Medicine in Hubei Province, Cooperative Innovation Center for Sustainable Pig Production, Wuhan 430070, China

**Keywords:** porcine reproductive and respiratory syndrome virus (PRRSV), sanguinarine, antiviral, alkaloid, target

## Abstract

Porcine reproductive and respiratory syndrome virus (PRRSV), the etiological agent of PRRS, is prevalent worldwide, causing substantial and immense economic losses to the global swine industry. While current commercial vaccines fail to efficiently control PRRS, the development of safe and effective antiviral drugs against PRRSV is urgently required. Alkaloids are natural products with wide pharmacological and biological activities. Herein, sanguinarine, a benzophenanthridine alkaloid that occurs in many plants such as *Macleaya cordata*, was demonstrated as a potent antagonist of PRRSV. Sanguinarine attenuated PRRSV proliferation by targeting the internalization, replication, and release stages of the viral life cycle. Furthermore, ALB, AR, MAPK8, MAPK14, IGF1, GSK3B, PTGS2, and NOS2 were found as potential key targets related to the anti-PRRSV effect of sanguinarine as revealed by network pharmacology and molecular docking. Significantly, we demonstrated that the combination of sanguinarine with chelerythrine, another key bioactive alkaloid derived from *Macleaya cordata*, improved the antiviral activity. In summary, our findings reveal the promising potential of sanguinarine as a novel candidate for the development of anti-PRRSV agents.

## 1. Introduction

Porcine reproductive and respiratory syndrome (PRRS), one of the major economically significant porcine diseases, has devastated the swine industry worldwide since the 1980s [1]. PRRS virus (PRRSV), the causative agent of PRRS, is an enveloped, positive-strand RNA virus classified within the family *Arteriviridae*, order *Nidovirales*. The genome of PRRSV is ~15 kb in length and consists of at least 10 open reading frames (ORFs), including ORF1a, ORF1b, ORF2a, ORF2b, ORF3-7, and ORF5a [2]. Currently, although substantial effort has been undertaken to control and prevent PRRS, the commercially available vaccines have shown limited efficacy, which mainly results from the immunosuppressive property of PRRSV, the highly genetic diversity of PRRSV genome, and the phenomenon known as antibody-dependent enhancement (ADE) during PRRSV infection [3,4]. Therefore, the development of novel antivirals against PRRSV is urgently needed.

Traditional Chinese medicines (TCMs) have gradually emerged as important sources of promising antiviral herbs [5]. The bioactive ingredients derived from TCMs, such as flavones, alkaloids, and terpenoids, have been demonstrated to exert antiviral activities against PRRSV through diverse mechanisms. For instance, (–)-Epigallocatechin-3-gallate (EGCG), the most abundant catechin in green tea, acts as an antiviral against PRRSV by inhibiting viral attachment and penetration as well as downregulating the expression of proinflammatory cytokines [6]. Moreover, matrine, an alkaloid extracted from *Sophora flavescens Ait*, has been proven to exhibit anti-PRRSV properties by targeting PRRSV replication and suppressing the secretion of IL-1β [7]. Additionally, as a common organosulfur constituent of *Persian shallot*, pyrithione antagonizes the replication, packaging, assembly, and release stages of PRRSV via multisite inhibition mechanisms [8].

Sanguinarine is a quaternary benzo[c]phenanthridine alkaloid that can be isolated from many plants, including *Macleaya cordata*, *Bocconia frutescens*, *Chelidonium majus*, *Poppy fumaria*, and *Sanguinaria canadensis* [9]. The anti-inflammatory, anti-tumor, and antimicrobial properties of sanguinarine have been reported extensively [10,11,12,13], while, to our knowledge, the antiviral activity has rarely been reported, with only a moderate observed antiviral effect of sanguinarine on tobacco mosaic virus (TMV) [14]. Recently, 8-hydroxydihydrosanguinarine, a derivative of sanguinarine, has also been demonstrated as a potential drug against COVID-19 [15]. Herein, we found that sanguinarine possessed a potent antiviral effect on the proliferation of PRRSV by targeting multiple stages of virus infection, including internalization, replication, and release processes. Mechanistically, through network pharmacology and molecular docking, ALB, AR, MAPK8, MAPK14, IGF1, GSK3B, PTGS2, and NOS2 were identified as potential anti-PRRSV targets of sanguinarine. Moreover, we improved the antiviral effect through the combination of sanguinarine with chelerythrine, another key bioactive alkaloid derived from *Macleaya cordata*. Our study provides a new candidate source for developing novel anti-PRRSV therapies.

## 2. Materials and Methods

### 2.1. Cells, Virus, and Reagents

Monkey kidney (MARC-145) cells were cultured in Dulbecco’s Modified Eagle’s Medium (DMEM) (Invitrogen, Waltham, MA, USA) supplemented with 10% fetal bovine serum (FBS). The HP-PRRSV strain WUH3 (GenBank Accession No. HM853673) isolated in 2006 by our laboratory [16] was amplified and titered in MARC-145 cells. Chelerythrine, sanguinarine, and BE60 (the drug with chelerythrine and sanguinarine as the primary active component) were gifted by Dr. Jianguo Zeng at Hunan Agricultural University, China [17].

### 2.2. Cytotoxicity Assay

The cytotoxicity of chelerythrine, sanguinarine, and BE60 was evaluated using CellTiter-Glo Luminescent Cell Viability Assay reagent (Promega, Madison, WI, USA). Briefly, MARC-145 cells in black glass-bottom 96-well plates were treated with chelerythrine, sanguinarine, and BE60 for 36 h, respectively. Then, CellTiter-Glo reagent (100 μL/well) was added, and the plates were shaken for 2 min. After incubation for another 10 min at room temperature, the cytotoxicity was determined using a 1450 MicroBeta TriLux instrument (PerkinElmer, Waltham, MA, USA).

### 2.3. The 50% Tissue Culture Infectious Dose (TCID_50_) Assay

MARC-145 cells seeded in 96-well plates were infected with serial 10-fold dilutions of samples in eight replicates. The plates were incubated for about 5 days at 37 °C, and the titers of PRRSV were calculated using the Reed–Muench method and are shown as the TCID_50_/mL.

### 2.4. Western Blot Assay

MARC-145 cells were washed twice with phosphate-buffered saline (PBS), and then lysed with RIPA Lysis Buffer (Beyotime, Shanghai, China) supplemented with protease inhibitor cocktail (Beyotime). The cell lysates were quantified with a BCA Protein Assay Kit (Beyotime), and denatured by boiling in 5× sodium dodecyl sulfate (SDS) loading buffer at 95 °C for 10 min. Equal amounts of proteins were subjected to SDS polyacrylamide gel electrophoresis (SDS-PAGE) and then electroblotted onto polyvinylidene difluoride (PVDF) membranes (Millipore, Darmstadt, Germany). Subsequently, the members were blocked with 10% (*w*/*v*) nonfat milk, and the proteins were probed with specific primary antibodies and HRP-conjugated secondary antibodies. The monoclonal antibodies (mAbs) against the nucleocapsid (N) protein and nonstructural protein 2 (nsp2) of PRRSV were prepared in our laboratory. The mAb against β-actin (AC026) was obtained from Abclonal Technology (Wuhan, China). The protein signals were detected using enhanced chemiluminescence (ECL) reagent (Bio-Rad, Hercules, CA, USA).

### 2.5. RNA Extraction

The total RNA was extracted using TRIzol reagent (Invitrogen). Briefly, cells (~10^6^) were lysed with 1 mL of TRIzol, followed by the addition of trichloromethane (200 μL) to each sample and subsequent thorough shaking. After centrifugation (4 °C) at 12,000 rpm for 10 min, the supernatant (400 μL/tube) was transferred to a new tube, then an equal volume of isopropanol (400 μL/tube) was added to each tube, gently mixed, and left for 10 min at room temperature. After another centrifugation (12,000 rpm, 10 min), the supernatant was discarded, and the RNA was washed twice with 1 mL of ethanol (75%) combined with two centrifugations (7600 rpm, 5 min). Finally, the residual ethanol was completely removed, with total RNA dissolved in DEPC.

### 2.6. Real-Time Reverse-Transcriptase Quantitative PCR (RT-qPCR)

To probe the effect of sanguinarine on the proliferation of PRRSV, the abundance of PRRSV ORF7 and nsp9 RNA transcripts was determined via RT-qPCR. Briefly, equal amounts of RNA were reverse transcribed into cDNA with oligo (dT) primers and Transcriptor First Strand cDNA Synthesis Kits (Roche, Basel, Switzerland). Then, the cDNA was used for qPCR in triplicates with primers (Table 1) specific to PRRSV ORF7 and nsp9 genes, respectively. The qPCR was conducted with Power SYBR green PCR master mix (Applied Biosystems, Waltham, MA, USA) in an ABI Q6 Flex real-time PCR system (Applied Biosystems).

To explore the role of sanguinarine in the replication stage of PRRSV infection, the PRRSV negative-sense RNA level was quantified as well. Especially, RNA was reverse transcribed into cDNA with 5′UF primers (Table 1), which bind to the viral negative-sense RNA, and the primers (Table 1) specific to PRRSV 5′UTR were used in qPCR.

### 2.7. Immunofluorescence Assay

MARC-145 cells cultured in black, glass-bottom 96-well plates were washed three times with PBS and then fixed with 4% paraformaldehyde (400 μL/well) for 15 min, followed by permeabilization with −20 °C precooled methanol (1 mL/well) for 10 min. Next, the cells were washed three times with PBS. The nuclei were dyed with DAPI. Finally, the fluorescence images and the PRRSV infection rate were obtained using the Opera Phenix high-content screening system (PerkinElmer).

### 2.8. Plaque Assay

MARC-145 cells seeded in 6-well plates were incubated with the serial 10-fold dilutions of viral samples for 1 h, then the cells were washed twice with PBS. The plaque fluid consisting of 1.8% low-melting-point agarose and DMEM supplemented with 6% FBS (1:1 *vol*:*vol*) was added to plates, and cells were cultured for 2~3 days. The plaque-forming units (PFU) were counted after staining cells with neutral red dye at 37 °C for 1 h. The data were obtained from three independent experiments.

### 2.9. Antiviral Assay

Taking sanguinarine as an example, MARC-145 cells were preincubated with sanguinarine for 2 h at 37 °C, followed by the substitution of supernatant with PRRSV, which was pretreated with sanguinarine for 1 h at 4 °C, at the multiplicity of infection (MOI) of 0.5 for another 2 h at 4 °C. Next, the supernatant was discarded, and the cells were washed twice with PBS, followed by incubation with sanguinarine for the indicated time. Finally, the TCID_50_ assay, Western blot assay, RT-qPCR, and/or immunofluorescence assay were performed to explore the potential anti-PRRSV activity of sanguinarine.

### 2.10. Attachment Assay

MARC-145 cells were precooled at 4 °C, then the cells were simultaneously incubated with PRRSV (MOI = 0.5) and sanguinarine (0.80 μg/mL) at 4 °C. Two hours later, the supernatant was discarded, and then, the cells were washed twice with PBS precooled at 4 °C, followed by the addition of plaque fluid. Finally, the number of plaques was counted after 2~3 days.

### 2.11. Internalization Assay

MARC-145 cells were precooled at 4 °C, then the cells were infected with PRRSV (MOI = 0.5) at 4 °C for 2 h to permit attachment. After two washes with PBS precooled at 4 °C, the cells were treated with sanguinarine (0.80 μg/mL) for another 3 h at 37 °C to permit internalization, followed by two washes with PBS. Finally, the plaque fluid was added, and the number of plaques was counted after 2~3 days.

### 2.12. Replication Assay

MARC-145 were infected with PRRSV (MOI = 0.5) for 6 h. Next, the cells were washed twice with PBS and incubated with sanguinarine (0.80 μg/mL) for another 1, 2, 3, and 4 h, respectively. The PRRSV replication level was determined by the amount of negative-sense RNA, which was measured via RT-qPCR.

### 2.13. Release Assay

MARC-145 were infected with PRRSV (MOI = 0.5) for 18 h. Next, the cells were washed twice with PBS and incubated with sanguinarine (0.80 μg/mL) for another 15, 30, and 45 min, respectively. The titer of PRRSV in the supernatant was tested via plaque assay.

### 2.14. Anti-PRRSV Target Screening

The 3D Conformer SDF file of sanguinarine chemical structure, which was obtained from the PubChem database (https://pubchem.ncbi.nlm.nih.gov/ (accessed on 4 November 2022)), was uploaded to the PharmMapper database (http://www.lilab-ecust.cn/pharmmapper/ (accessed on 6 November 2022)) to obtain the potential target genes of sanguinarine. Next, these genes were annotated using the UniProt Knowledgebase (UniProtKB). Moreover, the potential targets of PRRS were obtained from the Comparative Toxicogenomics Database (CTD). Finally, the anti-PRRSV targets of sanguinarine were selected from the intersection of sanguinarine targets and PRRS targets and then displayed through the Venn diagram plotted by http://www.bioinformatics.com.cn/ (accessed on 7 November 2022).

### 2.15. PPI Network

The anti-PRRSV targets of sanguinarine obtained in 2.1.4 were imported into the String database (https://string-db.org/ (accessed on 2 January 2023)) to obtain TSV files and then processed using Cytoscape 3.9.1 software to obtain the protein–protein interaction (PPI) network, followed by topological analysis to screen the core anti-PRRSV targets of sanguinarine according to the degree UnDir, betweeness centrality, and closeness centrality.

### 2.16. GO and KEGG Pathway Analysis

The anti-PRRSV targets of sanguinarine obtained in 2.1.4 were imported into the DAVID database (https://david.ncifcrf.gov/ (accessed on 7 January 2023)) for gene ontology (GO) and Kyoto encyclopedia of genes and genomes (KEGG) pathway analysis. The “select identifier” was set as “official gene symbol”, and the “list type” was set as “gene list”. Molecular function (MF), biological process (BP), and cellular component (CC) were included in GO analysis. *p* < 0.05 was considered significant. The top 10 GO enrichment results and top 20 KEGG pathways were selected according to the number of participating genes. The histograms for GO analysis and the bubble plots for KEGG analysis were plotted by http://www.bioinformatics.com.cn/ (accessed on 8 January 2023). Further topological analysis was conducted to build the network of sanguinarine-core target-pathway using Cytoscape 3.9.1 software.

### 2.17. Molecular Docking

The 3D Conformer SDF file of sanguinarine was converted to the Mol2 format, which served as a ligand. The 3D structure of ALB (PDB ID: 6OCL), AR (PDB ID: 4OHA), MAPK8 (PDB ID: 6ZR5), MAPK14 (PDB ID: 6SFO), IGF1 (PDB ID: 1WQJ), GSK3B (PDB ID: 6Y9S), PTGS2 (PDB ID: 3NT1), and NOS2 (PDB ID: 4NOS) were obtained from RCSB PDB database (https://www.rcsb.org/ (accessed on 10 January 2023)) and saved as pdbqt files, which were served as receptors. Subsequently, the IGEMDOCK software was used to dock the ligand and receptors, and then, the images were plotted through PyMoL software.

### 2.18. Statistical Analysis

GraphPad Prism 8 software (GraphPad Software, La Jolla, CA, USA) was used for data analysis using two-tailed Student’s *t*-test and one-way or two-way analysis of variance (ANOVA).

## 3. Results

### 3.1. Sanguinarine Exhibits Anti-PRRSV Activity

**Figure 1:**
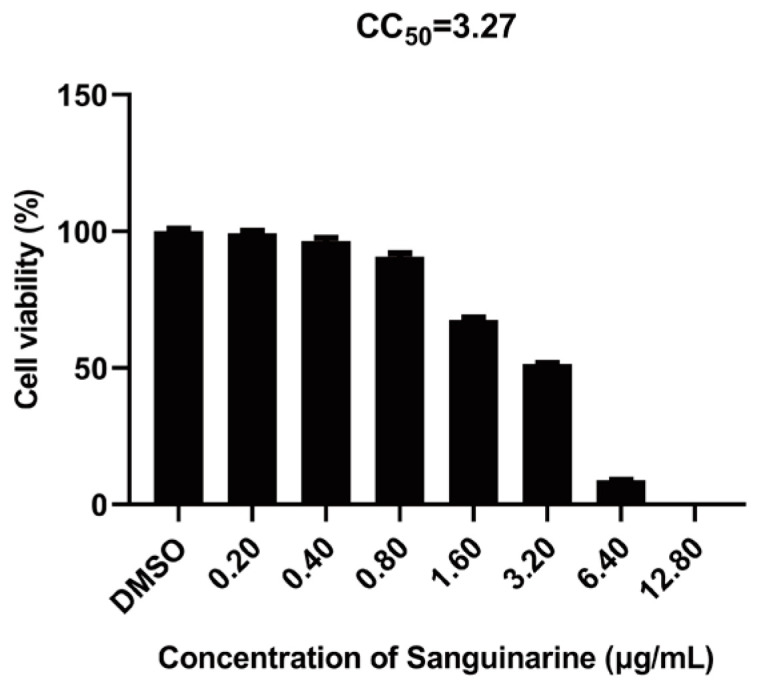
Cytotoxicity of sanguinarine on MARC-145 cells. MARC-145 cells were incubated with sanguinarine at different concentrations (0.20, 0.40, 0.80, 1.60, 3.20, 6.40, and 12.80 μg/mL) for 36 h; then, the cell viability was estimated using CellTiter-Glo Luminescent Cell Viability Assay. Data are expressed as the means from three independent experiments.

To determine the concentrations of sanguinarine for the subsequent experiments, the cytotoxicity of sanguinarine on MARC-145 cells was evaluated. As shown in Figure 1, the 50% cytotoxic concentration (CC_50_) of sanguinarine was 3.27 μg/mL, and no obvious cytotoxicity of sanguinarine on MARC-145 cells was observed when the concentration was lower than 0.80 μg/mL.

**Figure 2:**
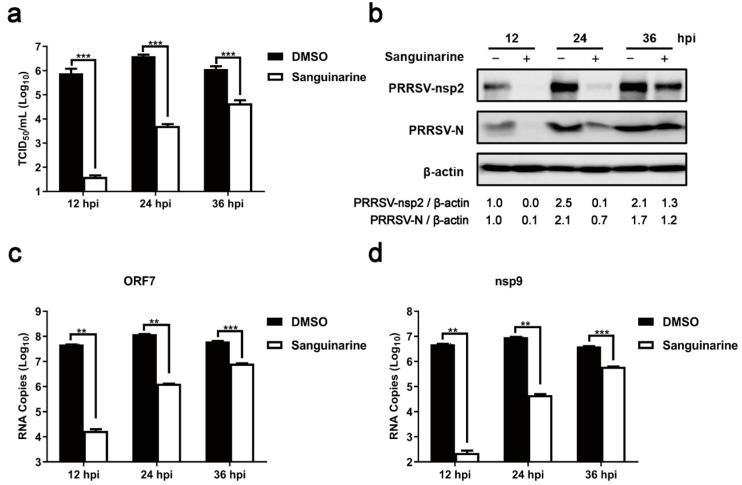
Sanguinarine significantly restricts PRRSV infection. MARC-145 cells were incubated with sanguinarine (0.80 μg/mL) and PRRSV (MOI = 0.5) as described in 2.9 of the Section 2. The group treated with DMSO was set as a negative control. Then, the viral titers, protein levels of PRRSV N/nsp2, and mRNA levels of PRRSV ORF7/nsp9 were evaluated by TCID_50_ assay (**a**), Western blot assay (**b**), and RT-qPCR (**c**,**d**) at 12, 24, and 36 hpi, respectively. (**b**) The ratios of nsp2/β-actin and N/β-actin were analyzed using ImageJ. (**a**,**c**,**d**) Data are expressed as the means and standard deviations from three independent experiments and analyzed using two-way ANOVA. **, 0.001 ≤ *p* < 0.01; ***, *p* < 0.001.

Then, the anti-PRRSV potential of sanguinarine was detected via TCID_50_ assay. As illustrated in Figure 2a, treatment with 0.80 μg/mL of sanguinarine caused ~10^4.3^-fold reduction in virus titers at 12 h post infection (hpi), with ~10^2.9^-fold and ~10^1.4^-fold at 24 hpi and 36 hpi, respectively. Moreover, the corresponding inhibitory effects of sanguinarine on the expression of viral proteins, including structural protein (N protein) and nonstructural protein (nsp2), were also demonstrated through Western blot assays (Figure 2b). Additionally, we found that sanguinarine decreased the levels of genomic RNA (nsp9 as a representative gene) and subgenomic RNA (ORF7 as the representative gene), with the inhibitory effects diminished over time (Figure 2c,d), which was in line with the above results of TCID_50_ assay and Western blot assay. Taken together, sanguinarine exhibited significant anti-PRRSV activity.

### 3.2. Sanguinarine Dose-Dependently Inhibits PRRSV Proliferation

**Figure 3:**
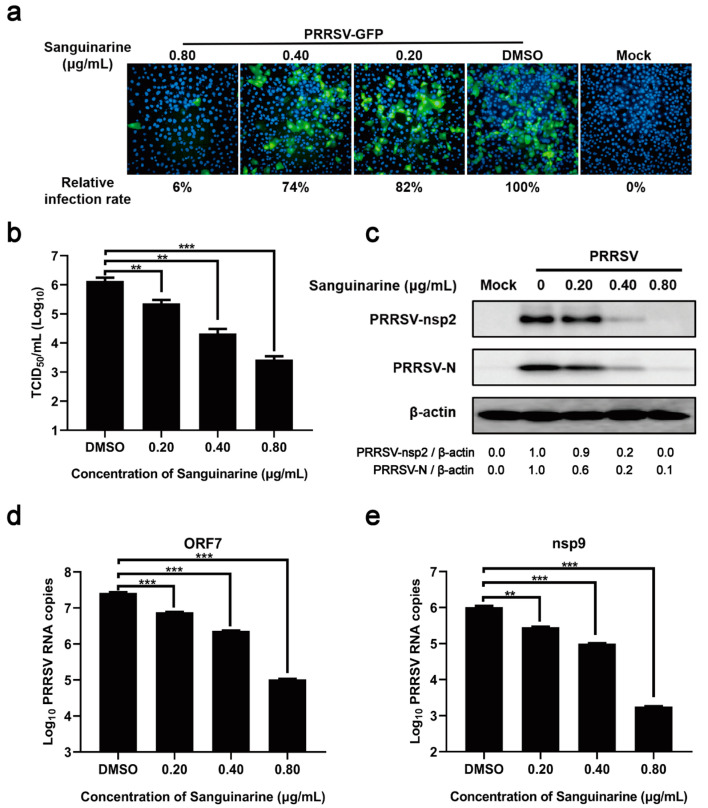
Sanguinarine inhibits the propagation of PRRSV in a dose-dependent manner. MARC-145 cells were incubated with sanguinarine (0.20, 0.40, and 0.80 μg/mL) and PRRSV/PRRSV-GFP (MOI = 0.5) for 24 h as described in 2.9 of the Section 2. The group treated with DMSO was set as a negative control. Then, the infection rate, viral titers, protein levels of PRRSV N/nsp2, and mRNA levels of PRRSV ORF7/nsp9 were evaluated by immunofluorescence assay (**a**), TCID_50_ assay (**b**), Western blot assay (**c**), and RT-qPCR (**d**,**e**), respectively. (**a**) The infection rate was averaged from nine visual fields. The infection rate of DMSO-treated group was set as 100%. (**c**) The ratios of nsp2/β-actin and N/β-actin were analyzed using ImageJ. (**b**,**d**,**e**) Data are expressed as the means and standard deviations from three independent experiments and analyzed using one-way ANOVA. **, 0.001 ≤ *p* < 0.01; ***, *p* < 0.001.

To further validate the inhibitory effect of sanguinarine on PRRSV infection, MARC-145 cells were treated with different doses of sanguinarine and infected with PRRSV-GFP (a PRRSV strain expressing green fluorescent protein) for 24 h. As shown in Figure 3a, when compared with DMSO-treated group, sanguinarine remarkably reduced the infection rate of PRRSV in a dose-dependent manner. Similarly, dose-dependent inhibitory effects of sanguinarine on the titers, protein expression levels (nsp2 and N), and genomic/subgenomic RNA levels (nsp9 and ORF7) of PRRSV were also confirmed through TCID_50_ assay and RT-qPCR, respectively (Figure 3b–e).

### 3.3. Sanguinarine Restrains PRRSV Infection via Multisite Inhibition Mechanisms

**Figure 4:**
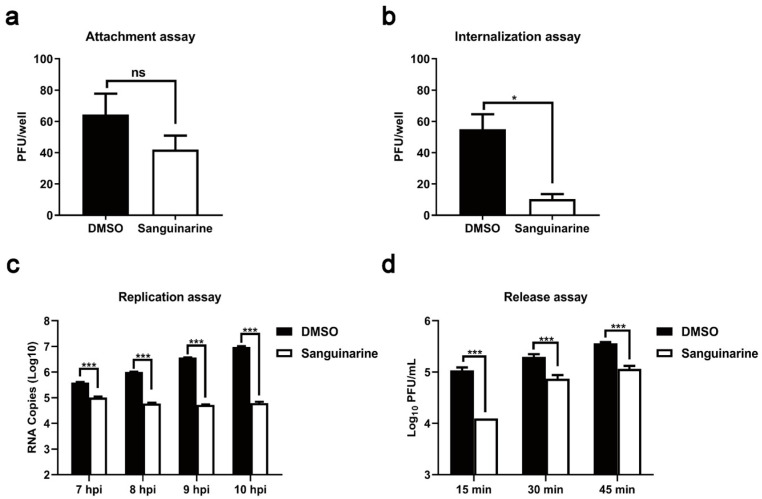
Sanguinarine exhibits anti-PRRSV properties by multisite inhibition mechanisms. MARC-145 cells were incubated with sanguinarine (0.80 μg/mL) and PRRSV as described in 2.10 (**a**), 2.11 (**b**), 2.12 (**c**), and 2.13 (**d**) of the Section 2. Data are expressed as the means and standard deviations from three independent experiments and analyzed using two-tailed Student’s *t*-test (**a**,**b**) or two-way ANOVA (**c**,**d**). *, 0.01 ≤ *p* < 0.05; ***, *p* < 0.001. ns, no significance.

To preliminarily pursue the underlying anti-PRRSV mechanism of sanguinarine, the effects of sanguinarine on each stage (attachment, internalization, replication, and release) of the viral life cycle were probed, respectively. Firstly, pre-chilled cells were incubated with PRRSV and sanguinarine (0.80 μg/mL) at 4 °C for 2 h to permit attachment but not internalization. Then, the influence of sanguinarine on the attachment process was detected via a plaque formation assay. As shown in Figure 4a, no significant inhibition on the number of viral plaques was observed under the treatment of sanguinarine. Secondly, pre-chilled cells were infected with PRRSV at 4 °C for 2 h and then treated with sanguinarine (0.80 μg/mL) at 37 °C for another 3 h; subsequently, a plaque formation assay was conducted to explore the effect of sanguinarine on the viral internalization stage. As illustrated in Figure 4b, the number of viral plaques in the sanguinarine-treated group was noticeably decreased compared with the DMSO-treated group. Thirdly, PRRSV-infected MARC-145 cells were incubated with sanguinarine (0.80 μg/mL) at 6 hpi; then, the level of negative-sense RNA was tested to determine the role of sanguinarine in regulating the replication stage of PRRSV infection. The results of RT-qPCR assays indicated that sanguinarine significantly downregulated the RNA copy numbers of PRRSV (Figure 4c). Fourthly, PRRSV-infected MARC-145 cells were treated with sanguinarine (0.80 μg/mL) at 18 hpi, and then, the total number of released progeny viral particles was measured using plaque assay to evaluate the effect of sanguinarine on the release process of PRRSV infection. As shown in Figure 4d, the production of progeny viruses was dramatically declined by sanguinarine. Altogether, sanguinarine inhibits internalization, replication, and release stages of PRRSV infection.

### 3.4. Network Pharmacology and Molecular Docking Analysis of Potential Anti-PRRSV Targets of Sanguinarine

**Figure 5:**
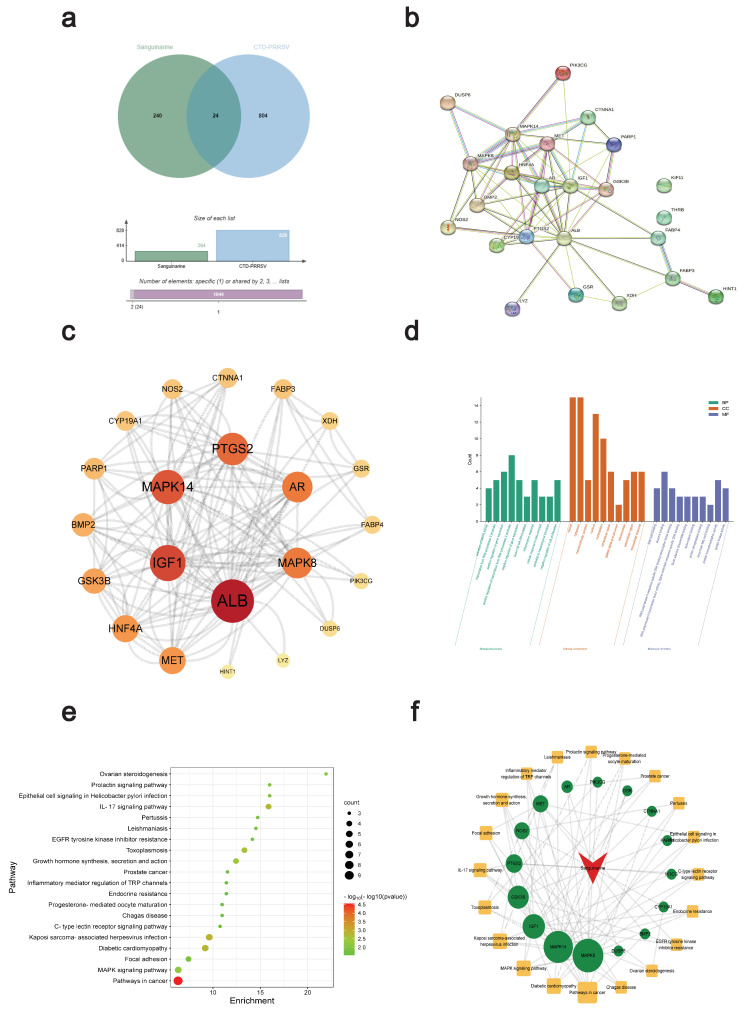
Screening of potential anti-PRRSV targets of sanguinarine through network pharmacology. (**a**) Intersection of sanguinarine targets and PRRS targets. (**b**) PPI network of the targets obtained in (**a**). (**c**) Topological analysis of the PPI network in (**b**). (**d**,**e**) Top 10 GO enrichment results (**d**) and top 20 KEGG pathways (**e**) of the targets obtained in (**a**). (**f**) Topological analysis of the network of the sanguinarine-core target-pathway.

**Figure 6:**
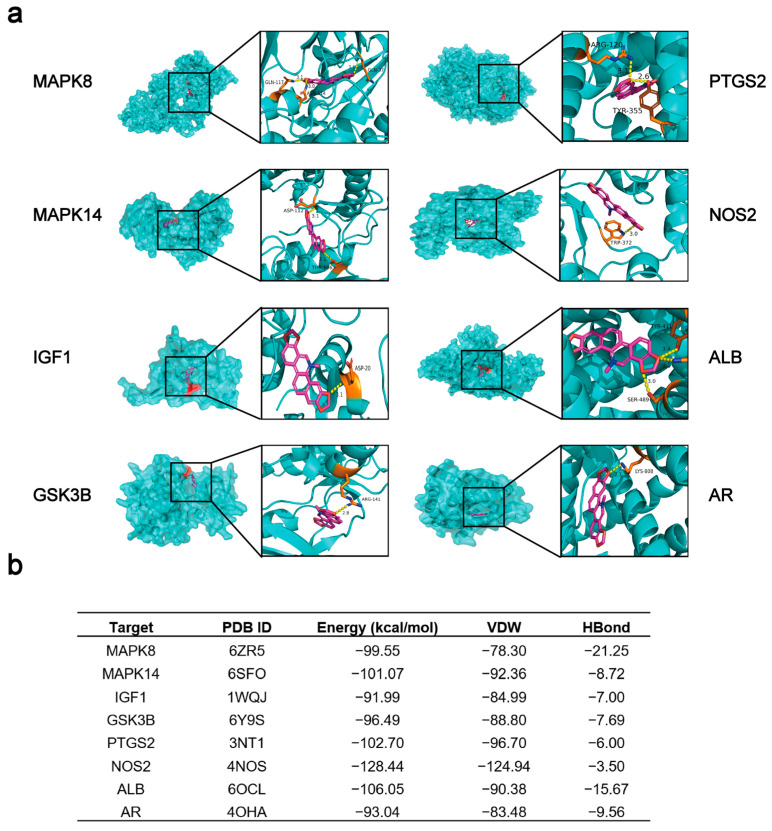
Molecular docking analysis of potential core anti-PRRSV targets of sanguinarine. (**a**) Schematic diagram of theoretical binding mode of sanguinarine and ALB, AR, MAPK8, MAPK14, IGF1, GSK3B, PTGS2, or NOS2. (**b**) The docking results of sanguinarine to ALB, AR, MAPK8, MAPK14, IGF1, GSK3B, PTGS2, or NOS2.

We first collected 264 potential targets (Table S1) of sanguinarine using the PharmMapper database and 828 PRRS targets (Table S2) using the Comparative Toxicogenomics Database (CTD), respectively. The intersection of the two groups of targets, a total of 24 targets, was selected for the following study (Table 2, Figure 5a). Subsequently, we constructed a protein–protein interaction (PPI) network of these 24 targets using the String database through Cytoscape 3.9.1 software (Figure 5b). With the degree UnDir ≥ 20, a further topological analysis of the obtained PPI network revealed a total of 6 core targets, including ALB (degree UnDir = 34), IGF1 (degree UnDir = 26), MAPK14 (degree UnDir = 24), PTGS2 (degree UnDir = 22), AR (degree UnDir = 20), and MAPK8 (degree UnDir = 20) (Figure 5c), which were chosen for further molecular docking analysis. Additionally, the gene ontology (GO) and Kyoto encyclopedia of genes and genomes (KEGG) pathway analysis of the above-mentioned obtained 24 targets showed 123 GO enrichment results and 41 pathways in total. The top 10 GO enrichment results and top 20 KEGG pathways that were sorted based on the *p*-value are illustrated in Figure 5d,e. Using the selected 20 pathways, we built the network of sanguinarine-core target-pathway, and the further topological analysis is displayed in Figure 5f. Then, with the degree UnDir ≥ 6, six core targets including MAPK8 (degree UnDir = 17), MAPK14 (degree UnDir = 16), IGF1 (degree UnDir = 11), GSK3B (degree UnDir = 10), PTGS2 (degree UnDir = 7), and NOS2 (degree UnDir = 6) were also chosen for further molecular docking analysis. We noted that the same four key targets (MAPK8, MAPK14, IGF1, and PTGS2) were obtained from the results of both Figure 5c,f. Subsequently, the key targets obtained from either Figure 5c,f, namely MAPK8, MAPK14, IGF1, PTGS2, ALB, AR, GSK3B, and NOS2, were all used for the molecular docking analysis. We observed that sanguinarine was well docked into the protein-binding pockets of these eight targets (Figure 6a). The docking results are shown in Figure 6b.

### 3.5. Combination with Chelerythrine Improves Antiviral Activity of Sanguinarine

**Figure 7:**
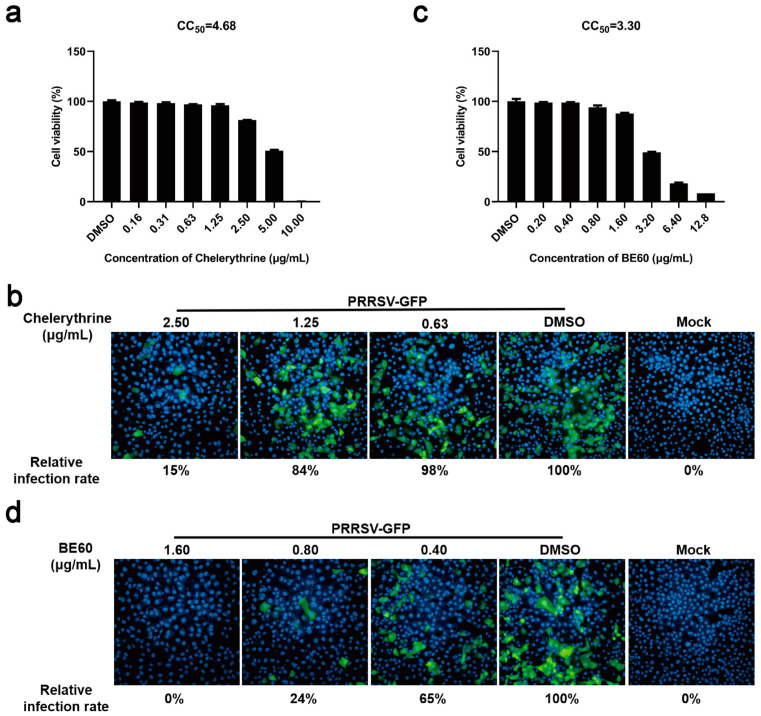
Combined use of sanguinarine and chelerythrine exerts potent antiviral efficacy against PRRSV. (**a**,**c**) MARC-145 cells were incubated with chelerythrine (**a**) at different concentrations (10.00, 5.00, 2.50, 1.25, 0.63, 0.31, and 0.16 μg/mL) or BE60 (**c**) at various concentrations (25.60, 12.80, 6.40, 3.20, 1.60, 0.80, and 0.40 μg/mL) for 36 h, respectively. Then, the cell viability was detected using CellTiter-Glo Luminescent Cell Viability Assay. Data are expressed as the means from three independent experiments. (**b**,**d**) MARC-145 cells were incubated with chelerythrine (**b**) (2.50, 1.25, and 0.63 μg/mL) or BE60 (**d**) (1.60, 0.80, and 0.40 μg/mL) and infected with PRRSV (MOI = 0.5) for 24 h as described in 2.9 of the Section 2. The group treated with DMSO was set as a negative control. Then, the infection rate of PRRSV was determined by immunofluorescence assay, which was averaged from nine visual fields. The infection rate of the DMSO-treated group was set as 100%.

Chelerythrine is another key bioactive alkaloid obtained from *Macleaya cordata* [18]. The influence of chelerythrine on PRRSV infection was also preliminarily explored here. Firstly, the cytotoxicity of chelerythrine on MARC-145 cells was measured, which revealed that the CC_50_ of chelerythrine was 4.68 μg/mL, and the chelerythrine at the concentration under 2.50 μg/mL caused n-o obvious cytotoxicity to MARC-145 cells (Figure 7a). Then, MARC-145 cells were treated with different doses (0.63, 1.25, and 2.50 μg/mL) of chelerythrine and infected with PRRSV-GFP for 24 h. As shown in Figure 7b, the infection rate of PRRSV-GFP was strikingly downregulated by chelerythrine in a dose-dependent manner.

Combination has been shown to enhance therapeutic efficacy. Therefore, the antiviral effect of the sanguinarine–chelerythrine combination was explored. MARC-145 cells were treated with different concentrations of the mixture with sanguinarine and chelerythrine (2:1) as the predominant active ingredients, which is abbreviated hereafter as BE60. The result of cytotoxicity assay revealed that the CC_50_ of BE60 was 3.30 μg/mL, and BE60 at the concentration below 1.60 μg/mL exhibited no obvious cytotoxicity on MARC-145 cells (Figure 7c). Then, the antiviral potential of BE60 at different concentrations was tested, which showed that BE60 dose-dependently inhibited PRRSV-GFP infection, with no PRRSV-infected cells observed when treated with BE60 at the highest safe concentration (1.60 μg/mL) (Figure 7d). Of note, the PRRSV-infected cells still existed in the groups treated with sanguinarine or chelerythrine alone at the highest safe concentration. The above data suggest that the combination between sanguinarine and chelerythrine seem promising, with an increase in the anti-PRRSV activity.

## 4. Discussion

Currently, numerous antiviral agents against PRRSV have been identified [19]. Among them, traditional Chinese medicines (TCMs) have attracted increasing attention due to their wide varieties of forms, low drug resistance, low price, and low toxicity/side effects [20,21]. TCMs exert anti-PRRSV activity through multiple mechanisms including inhibition effects on viral life cycles (attachment, internalization, replication, and release), and modulation of immune responses and pathogenic pathways [22]. Our study revealed that sanguinarine, a natural plant-derived bioactive compound, acts as an antiviral against PRRSV by targeting the internalization, replication, and release stages but not attachment process. Additionally, other significant bioactive characteristics of sanguinarine, such as its anti-inflammatory properties, have been proven previously [23]. Noteworthily, many TCMs exert antiviral effects on PRRSV via inhibition of the inflammatory response. For example, Proanthocyanidin A2 (PA2), one bioactive extract of grape seed *Vitis vinifera*, acts as an antagonist of PRRSV, partly by deactivating the NF-κB pathway [24]. Matrine, EGCG, and *Sasa quelpaertensis Nakai* extracts significantly reduce the levels of proinflammatory cytokines such as IL-6 and IL-8, which contribute to the suppression of PRRSV infection [6,7,25]. Therefore, the anti-inflammatory activity of sanguinarine may account for its anti-PRRSV property, which requires additional trials to verify.

Sanguinarine along with chelerythrine are known as main effective alkaloids of *Macleaya cordata* [26]. Sangrovit^®^ is a low-cost, safe, and effective feed additive derived from *Macleaya cordata*, with sanguinarine and chelerythrine as the major bioactive compounds [27]. The beneficial effects of sangrovit^®^ on the growth performance and gut health of piglets, broiler chickens, and fishes have been verified [28,29,30]. Moreover, sangrovit^®^ exerts significant and broad-spectrum antimicrobial activity and thus has been used as an antibiotic alternative [31]. Here, we demonstrated the anti-PRRSV property of sanguinarine and chelerythrine, which provides further evidence for the significance of the application of sangrovit^®^ in the pig-feed industry.

In addition to sanguinarine and chelerythrine, allocryptopine and protopine are the other two key alkaloids extracted from *Macleaya cordata* [32]. The insecticidal, antiseptic, anti-tumor, and anti-inflammatory activity of extracts of *Macleaya cordata* has been reported previously, with the above four alkaloids as major bioactive compounds [33]. Herein, the anti-PRRSV activities of allocryptopine and protopine were also observed (Figure S1). Notably, the anti-PRRSV effects of these four alkaloids extracted from *Macleaya cordata* were limited, especially in the late stage of PRRSV infection. Therefore, it is of significance to improve their antiviral activity through effective strategies. In our group, a set of compounds from sanguinarine has been constructed using the complexity-to-diversity (CtD) approach, according to previous studies [34,35,36]. The antiviral potential of these sanguinarine-derived compounds will be detected in the future to achieve antivirals with superior anti-PRRSV activity.

In summary, our findings demonstrate the antiviral effect of sanguinarine on PRRSV and reveal the underlying mechanism by targeting viral internalization, replication, and release stages. We also revealed the potential anti-PRRSV targets of sanguinarine, including ALB, AR, MAPK8, MAPK14, IGF1, GSK3B, PTGS2, or NOS2, through network pharmacology and molecular docking, which provides important insights for future anti-PRRSV studies involving sanguinarine. Moreover, we found that combination with chelerythrine improves the anti-PRRSV activity of sanguinarine. Overall, this study proves the potential of sanguinarine as a novel candidate for future antiviral research.

## Figures and Tables

**Table 1:** The sequences of primers used in this study.

Name	Forward Sequence (5′-3′)	Reverse Sequence (5′-3′)
nsp9	ACCCTAGGACCTGTGAAC	GGCGAGTAACTTAGGAGATG
ORF7	GCAATTGTGTCTGTCGTC	CTTATCCTCCCTGAATCTGAC
5′UTR	GCATTTGTATTGTCAGGAGC	AGCAGTGCAACTCCGGAAG
5′UF	GACGTATAGGTGTTGGCTC	

**Table 2:** The potential anti-PRRSV targets of sanguinarine.

Names of Target Genes
BMP2, AR, ALB, CYP19A1, LYZ, GSR, KIF11, DUSP6, NOS2, PTGS2, CTNNA1, THRB, MAPK14, HNF4A, MET, MAPK8, PARP1, GSK3B, IGF1, PIK3CG, XDH, HINT1, FABP4, FABP3

## Data Availability

The datasets generated for this study are available on request to the corresponding author.

## Supplementary Materials

The following supporting information can be downloaded at: https://www.mdpi.com/article/10.3390/v15030688/s1, Table S1: The targets of sanguinarine predicted by the PharmMapper database, Table S2: The targets associated with PRRS predicted by the Comparative Toxicogenomics Database, Figure S1: Allocryptopine and protopine dose-dependently antagonizes PRRSV infection.
