# Supplementary material for: Sanguinarine Exhibits Antiviral Activity against Porcine Reproductive and Respiratory Syndrome Virus via Multisite Inhibition Mechanisms
Source: Viruses. 2023 Mar 6;15(3):688. doi: 10.3390/v15030688 (PMC10052745; doi:10.3390/v15030688)
Supplement: Supplementary file 1 [file viruses-15-00688-s001.zip › viruses-2263774-supplementary.pdf]

Table S1. The targets of sanguinarine predicted by PhamMapper database.

Names of target genes
PTGS1, BMP2, ATPE, NFT-1, AMPC, CCNA2, RHO, MUP2, DPS2, TESA, AR, PIM1, OMPF, 10, MUP1, ESR1, CAPNS1, KSI, RPLA, PLDA, BOP, OMPA, NSPA, APOA2, AKR1C2, ALB, RPH, CBL4, AKR1C9, STS, BTUB, YHAI, NR1I3, PPIA, CA2, CHI1, SOP2, CYP2B4, ESRRG, FABB, E, NR1H2, PGR, CYP19A1, LYZ, MMP13, SULT2A1, AKR1C3, PLA2G1B, ERYF, DPS, PDAD, GFRA1, ADAM17, FRR, GSR, BCHE, NOS3, CHEK1, THI1.2, THYA, UVRB, PTPN1, ESR2, SRC, PH1313, PPP5C, SHBG, NALP, ILVA, FECA, FOLB, HNL, ACCC, COBT, RADA, CDK2, PRX2, AKR1B1, FHUA, KIF11, DUSP6, HSD17B10, MXIM, FABI, PRSS1, CFD, RXRA, APX1, NUDC, NQO2, NR3C2, CUT1, OMPT, CAMC, PDE10A, LYTA, PRKACA, FBPB, SORD, GDI1, PDE4D, NOS2, BACE1, ACADM, METG, PTGS2, HSD17B1, GC, FABH, IMPDH, CYP102A1, F2, MAPK10, OPMA, EFT1, TRXA, POT1, SEC14, SSO2706, ACE, TGFBR1, PABC, XYLA, PHP, CELA1, ALPHA-MAN-IIA, PDE3B, HSP90AA1, TM_1620, TTR, CHS2, FBPC, PGF, F10, YECD, KDR, AKR1C1, SDH1, CTNNA1, MMP3, TYRB, ARGI, MMP8, PTPN11, PANC, TGT, HSD11B1, PAGP, THRB, BLA, ANXA5, MAPK14, LYSA, PAH, HNF4A, SHC, MURD, AKR1C5, MET, PDPK1, ADH1C, CH2, TRI5, FNTA, DEOD, LTP, YARS1, MAPK8, TTPA, CAT3, CROT, EPHB4, BPHC, PYRG, ANSB, YKOF, MGSA, ADH5, PARP1, PYGM, GAG-POL, RORB, MBTK, ADK, CDK6, CYP51, MMP12, EPHX2, PRSS2, ASPC, AVD, DPP4, SEC14L2, PNP, GPD, DHODH, KYNU, LPXC, ZIPA, GSK3B, HDAC8, NMNAT1, OPR1, TESB, SERPINA1, DCD, CMAA1, GRIA2, SULT1E1, KYAT1, IMPDH2, GABT, LTP1, VCP, SDHA, RNASE1, PRMC, PGIA, MRE11, SUFS, BIOA, HPD, NR1H3, IIGP1, IGF1, ACP3, MHCA, FADL, PHZD1, ADA, ADF1, PKIA, MTHFD1, PLC, ERBB4, PPT1, GM2A, PDE4B, PIK3CG, BLAB1, XDH, BIOD1, RBP4, AHRI, LIP, GAPA, MAOB, DEF, FABA, KDSA, GSTA1, HINT1, ALR, TRPA, DHFR, FABP4, NA, LEUB, SULT2B1, FABP3, FKBP1A, MMAA2, TM_1468

Table S2. The targets associated with PRRS predicted by Comparative Toxic genomics Database.

Names of target genes
IFNB1, IL12B, IFNG, RELA, IL1B, IL12, IL6, TNF, LGR1, IL1B2, TLR7, ATP1A1OS, IFIT1, LYRIC, IRF7, ISG15, SRP14-DT, RN28S1, RN18S, WASHC2, SHOX, ZNF354C, HBA, MNDAL, FOXO3A, VLDLR-AS1, LCN1, GALP, NIBAN3, ZNF777, ZCCHC4, GALR3, IL1F10, ABRAXAS2, DAF-16, C18ORF25, WDR20, PEA15A, PODNL1, STRIP1, EMC10, H1F5, HEMGN, SLC4A1AP, USP30, SPRR3, ZBTB33, NPFFR2, ALKBH1, KLHL30, RNF111, CDAN1, CERCAM, SPATA5L1, WRAP53, GPN1, DNAH10, HOXA7, ZBED8, ARMC10, RPP30, TXNDC11, ACTBL2, ADIRF, P4HA3, TOMM70A, TRMT13, CHAMP1, HINFP, IL22RA2, CWF19L2, MEIS3, TBP, ANGPTL7, BEND7, CCDC112, EID2, IL21, SETDB1, SH3BP1, ZBTB11, ATP5C1, CASC3, ENGASE, FBXO28, HBB-B1, IST1, MRPL39, MUC16, RAVER1, RBBP5, SLC25A6, TOMM22, AK7, DSC1, GDF5, GLDN, PHAX, SNW1, GPKOW, IFNAR1, KHDC4, RALY, SMARCC2, CD248, FMNL1, MZF1, SRFBP1, XCR1, CCL25, MTREX, PLRG1, SETD1A, ITGA10, LOXL3, NRROS, PI4KB, ANKRD13C, CYTL1, METTL14, MRPS30, MRPS31, HAUS2, IL36G, NDUFB7, SRSF9, B3GALT4, CACNG6, CRELD1, IL9, MRAP2, NUFIP1, PDSS2, RAE1, SCLT1, SNIP1, TFAP4, USP39, WBP4, WDR5, WDR74, ASCL2, CNPY2, CYP4F3, DDX52, DYNC1LI1, GALR2, GIP, IL18RAP, MRPS22, UTP3, HYLS1, MIR17HG, MTA3, NUDCD1, SFT2D2, TFF2, CCNY, CPSF2, OSTF1, PDCD1, SH2D3C, TMBIM6, ZBTB18, ATP6V1H, BNIP1, FBXO22, KAT2A, MIR221, RCOR1, ARGLU1, ATG14, ATP10A, CBLL1, CORO2B, CTSO, FASTKD2, HLX, ITGBL1, KRT20, PLPP5, PRPF38B, RBM17, RHOH, WFDC1, BNIP2, GHRH, PDE12, RANBP2, SEC61B, UGT2B4, VAMP4, FUT1, GP1BB, NDUFB9, PCYOX1L, UPF2, AGFG1, BUB3, GKAP1, MALL, RAB18, ATP6V1G1, CARD11, DNAJC5, GABPA, H1-10, RNPS1, SF3A1, SYNPO2, UNC13D, ZC3H11A, KHDRBS3, PTPN22, ATG4B, ATP8B2, G3BP2, GATAD2A, PRKD2, RBM12, RBM15, RPS29, SSRP1, THRAP3, USP24, ATP5H, DNTTIP2, NANOS1, NDUFS7, PDLIM4, PSTPIP2, RICTOR, CCR4, DNAJC1, KCNMB4, LMO2, NOD2, SAFB, SGPP1, SRRM1, TXNDC16, BST1, CHN1, CKAP5, EIF2B4, HAPLN1, OSER1, ASCC3, BRD3, H1-0, IFNA1, KBTBD11, MYO5C, PLEKHO2, PYY, TM9SF3, ZBTB21, CCL24, MICALL1, NAGLU, NUP155, SOX5, SYBU, TBC1D15, UQCRFS1, ALPK2, BPTF, CCR3, EMILIN1, FABP6, IL22, LARP7, NDUFS5, PPP3CB, PTPN14, PTPRJ, RNASET2, SUV39H2, ACAN, ALPI, FMOD, KDM4B, NDUFV3, NIPBL, SP100, TFCP2L1, UPF3B, VIL1, ACIN1, AKAP8L, ATF1, CLIC3, CYP21A2, GALNT1, H2BC5, PDCD11, ABI1, BACH2, BCL2L13, EP400, IL10RB, MUC5B, NUMA1, NUP98, PLEKHA6, PRR5L, RCC1, RPLP2, RSL1D1, TCOF1, CCL1, CTHRC1, NFYA, PRELP, RBM25, TOMM40, USP15, AJUBA, ARHGAP45, BIN1, CCL12, EPB41L2, EVI2A, EXOSC2, FUCA1, HINT1, ITIH5, SCARA5, CYC1, HDGF, MAPK12, MUC2, NDUFB8, PSPC1, RPL4, RPLP1, TLR5, C3AR1, CCR6, CXCR1, EEF1B2, EIF5B, EVL, RUVBL2, SEMA3A, SPTLC2, CASR, MMUT, MSX2, PRKAR2A, RPL22L1, SPRR1A, STRBP, TNFSF14, TRIM28, CGREF1, EIF3C, LGR5, RPL27A, SF1, STEAP3, DEFB1, PDLIM2, PRKCG, ATP6V1B2, BMI1, CENPW, COL15A1, EIF3B, LPXN, TFF3, KDELR3, MDM4, NUCKS1, OIP5, PCOLCE, RBBP6, ALDH3B1, AMIGO2, COX5B, MRPS18B, NDUFS4, OAS2, RBM39, RPS6KA2, SLC10A2, SORBS2, WWTR1, ATP6V1A, ATP6V1D, CBX5, FNBP1, H1-2, MAP4, RPS6KA5, SYNM, DDX39A, MKNK2, MYH11, PRKAR1A, TOMM20, ABCF1, EEF1D, ELAVL1, LTBP2, PSIP1, SAMHD1, BCL10, DFFA, ECM1, ETNK1, FAM83D, G3BP1, IGFALS, IRF2BP2, LTA, MYBBP1A, RAMP1, RPL31, RPLP0, SLC51A, TLR1, CACNA1D, DKC1, EIF2S2, HCK, LIPA, PALLD, PIK3CG, PSMD12, SERPINA3, SLC4A7, SRRM2, STAT4, SULF1, TP53INP1, ANKRD37, CCL17, FASL, INPP5D, LYZ, PPM1D, RIPK2, UQCRC1, C4B, CREB5, DUSP8, KAT2B, LSP1, BCLAF1, CLDN2, DDB2, IL7, NOP56, RGS5, SH3KBP1, SRSF2, WIPI1, CDH13, CKS1B, COL6A2, CTNNA1, DEPTOR, EIF4G1, ENG, FOXP3, GREM1, OGA, PGRMC1, PLEC, RPS6KA1, SRSF1, TOP2B, TSLP, CCL22, CORO1A, SHCBP1, STAT2, TRAF1, CD69, CXCL11, DDX21, IFNGR1, NOLC1, NR5A1, HSPA1L, IFITM3, RBM3, RRM2B, CLDN3, CYP11B1, EIF2A, KMT2A, MFN1, TRAF6, CCR1, CHKA,

CHORDC1, HDAC2, LAMB1, LCP1, LGALS3BP, MAP2K4, MYH9, PPP2CA, REL, SMOX, TNFSF9, TNS1, ALDOB, CKAP2, ELK1, LTB, MAP1LC3A, PSMB8, SPAG5, TJP2, CCR7, CISH, CLDN5, EIF2AK2, NCL, YAP1, ANLN, CXCR2, IL15, LHCGR, LIG1, OPA1, SDC1, CCK, DNAJC3, GCG, IRAK1, MYCN, NEDD4L, SOCS1, WARS1, ATRX, CXCL3, ECT2, IGFBP6, AXIN2, CANX, RGCC, SLC1A5, SLC9A3R1, TLR3, TMPO, TRPA1, TTK, AHNAK, LAMP2, NDC80, NFATC1, PML, PROS1, ZEB2, ANPEP, PHLDA3, RGS4, SOX4, TAP1, ZEB1, CYP11B2, FLNA, ACSL4, EIF4E, IL23A, KIF2C, MUC5AC, NFKBIB, RELB, VCAN, IL1RL1, NUSAP1, RORA, CD55, CD80, CLDN4, EZH2, HSD3B2, IL11, KIF11, MARCKS, MTHFD2, PRC1, XRCC1, COL5A1, FABP3, JAK1, KITLG, NPM1, SERPINE2, UGT2B7, UHRF1, ACTA1, CX3CL1, DNAJA1, FOSL2, GHR, BUB1B, CBS, CCL11, GH1, MFN2, NR4A3, ATF2, BCL6, NFKB2, SOCS2, BAG3, CCR2, CDKN3, IL33, LMNB1, HNF4A, MAP2K6, NR1D1, PTGES, CXCL5, CXCL9, HDAC1, MAP3K5, SLC16A1, TGFB3, UBE2C, CHAC1, COL4A1, PRKCE, CCL7, CCNG2, S100A4, EP300, FOSB, PCK2, PHLDA1, PSAT1, CD68, CREBBP, DNMT3A, ITGB2, NAMPT, JUND, PHGDH, FDPS, NTRK2, FDFT1, LMNA, STMN1, CCNG1, CLDN1, HSPH1, KLF4, NPY, OGG1, TFF1, TIMP3, CCL20, CHUK, BMP2, CSF3, GPX4, HSP90AB1, CD40, GPX3, IL1R1, ATF6, GPX2, PLIN2, ID1, SP1, TF, CYP1A, DUSP6, FOXO1, KRT18, IL12A, IL17A, MAFF, RPS6, TLR2, C3, CCN1, MYD88, OCLN, SOCS3, ACTB, BBC3, CD14, CXCR4, IL1RN, JAK2, BAK1, COMT, DNMT1, ERN1, IL13, NR4A1, TJP1, CCL4, EIF4EBP1, FOSL1, IGFBP3, IL5, MET, NLRP3, PMAIP1, THRA, DDIT4, PRKCA, CRP, ASNS, CD86, JUNB, CEBPA, KEAP1, TRIB3, F3, HSPA8, ODC1, FSHB, SLC7A11, RPS6KB1, THRB, FABP4, XBP1, XDH, BAD, BECN1, CYP11A1, DUSP1, MAPK14, PTEN, CCL3, MDM2, STAT1, ABCC3, CCNE1, MAP1LC3B, BID, CEBPB, EPHX1, CXCL10, STAR, CYP17A1, EGF, IL18, SIRT1, MKI67, CCL5, TRP53, CXCL2, CYP19A1, HMGCR, MAPK9, ATF3, SPP1, CXCL1, FN1, GADD45A, TLR4, CASP7, CDK1, CREB1, GCLM, GPX1, CYCS, MTOR, VIM, IL2, EGR1, IGF1, CCNB1, GSK3B, CTNNB1, FAS, IL1A, SQSTM1, PCNA, HIF1A, HSPA5, IL4, GPT, NFKB1, EGFR, MPO, STAT3, BCL2L1, CYP1B1, GSR, MAPK8, MYC, ABCB1, ICAM1, DDIT3, ALB, IL10, SOD1, JUN, AR, CASP8, NFKBIA, NQO1, SOD2, FOS, TGFB1, CCND1, CYP3A4, PPARG, CCL2, CDKN1A, NFE2L2, CXCL8, PARP1, CASP9, AKT1, NOS2, TP53, CYP1A1, HMOX1, CAT, PTGS2, BAX, BCL2, MAPK1, MAPK3, CASP3

Figure S1

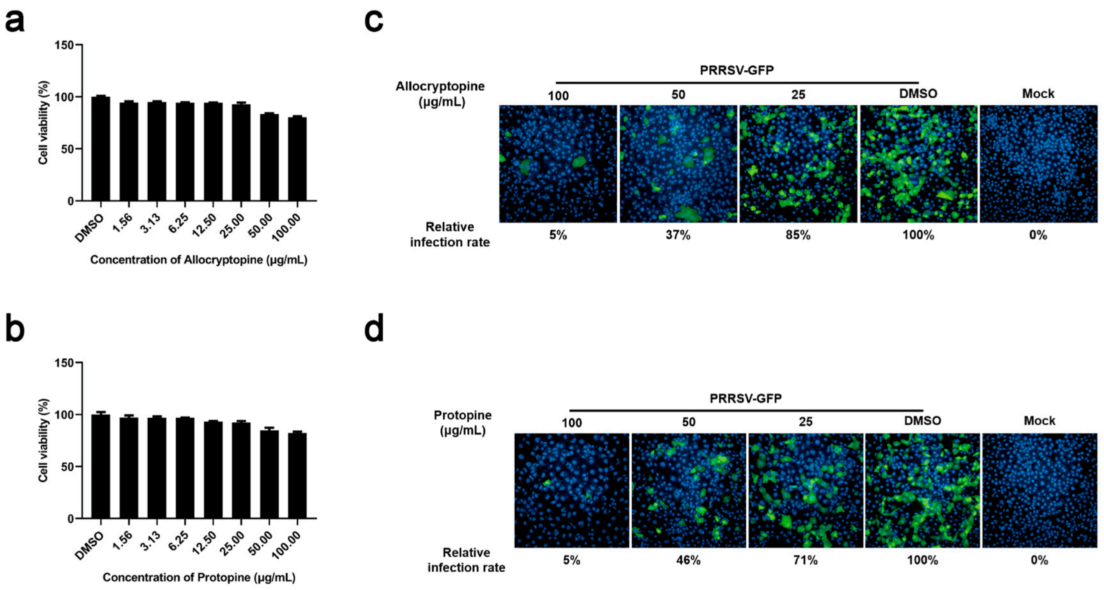

Figure S1. Allocryptopine and protopine dose-dependently antagonize PRRSV infection. (a,b) Cytotoxicity of allocryptopine (a) or protopine (b) on MARC-145 cells. MARC-145 cells were incubated with allocryptopine (a) or protopine (b) at different concentrations for 36 h, then the cell viability was estimated using CellTiter-Glo Luminescent Cell Viability Assay. Data are expressed as the means from three independent experiments. (c,d) MARC-145 cells were incubated with allocryptopine (c) or protopine (d), and infected with PRRSV-GFP (MOI = 0.5) for 24 h as described in Figure 3a. The group treated with DMSO was set as a negative control, and the infection rate of DMSO-treated group was set as 100%. The viral infection rates were evaluated by immunofluorescence assays, and each infection rate was averaged from nine visual fields.
